# Supplementary material for: Sequestration of host metabolism by an intracellular pathogen
Source: eLife. 2016 Mar 16;5:e12552. doi: 10.7554/eLife.12552 (PMC4829429; doi:10.7554/eLife.12552)
Supplement: Supplementary file 1. — DOI: http://dx.doi.org/10.7554/eLife.12552.023 [file elife-12552-supp1.docx]

**Supplementary Table 1 ⏐ Primers used for cloning purposes.**

| Name | Vector | Primers |
| --- | --- | --- |
| glgC | pUC19cya | AGTCAAGCTTGTAATAGTTTTGTTTTTATGGCTGGTAGACGGACG |
|  |  | AGTCTCTAGATCCGCATAAGACAATAACTCCTAC |
| glgA | pUC19cya | AGTCAAGCTTGTAATAGTTTTGTTTTTATGAAAATTATTCACACAGCTATCG |
|  |  | AGTCTCTAGATGCTAGTCCGTATAGCGCG |
| glgB | pUC19cya | AGTCAAGCTTGTAATAGTTTTGTTTTTATGGATCCTTTTTTCTTAAATACTCAAC |
|  |  | AGTCTCTAGAAGAAACAATTCCCAAAAGATCTTG |
| glgP | pUC19cya | AGTCAAGCTTGTAATAGTTTTGTTTTTATGTATTTCGATCGGACAAAG |
|  |  | AGTCTCTAGATTGAGGAGTCTGGACTACCC |
| glgX | pUC19cya | AGTCAAGCTTGTAATAGTTTTGTTTTTATGGAATCTTTGTCTGTTCGTT |
|  |  | AGTCTCTAGAAGAAAATAGAGAAAAACGGTAGCG |
| malQ | pUC19cya | AGTCAAGCTTGTAATAGTTTTGTTTTTATGCCGTCATTATCCCAAT |
|  |  | AGTCTCTAGAGCCATGCTTAGGAGAAGTATCTATC |
| mrsA | pUC19cya | AGTCAAGCTTttaggttctgatcggaaggttgtctt |
|  |  | AGTCTCTAGAcaaaatgtttttggctgtttga |
| glgA | pCiNeo | AAAAAAGCAGGCTTCATGAAAATTATTCACACAGCTATCG |
|  |  | AGAAAGCTGGGTCTCATTATTGTTTATAAATTTCTAAATATTTATTGG |
| glgA | pDEST15 | AAAAAAGCAGGCTTCATGAAAATTATTCACACAGCTATCG |
|  |  | AGAAAGCTGGGTCTCATTATTGTTTATAAATTTCTAAATATTTATTGG |
| glgA*(E. coli)* | pGEX | GGATCCCAGGTTTTACATGTATGTTCAGAGATGTTCCCGCTGCTTA |
|  |  | CTCGAGCTATTTCAAGCGATAGTAAAGCTCACGGTACGACTT |
