# Supplementary material for: Sequestration of host metabolism by an intracellular pathogen
Source: eLife. 2016 Mar 16;5:e12552. doi: 10.7554/eLife.12552 (PMC4829429; doi:10.7554/eLife.12552)
Supplement: Supplementary file 2. — DOI: http://dx.doi.org/10.7554/eLife.12552.024 [file elife-12552-supp2.docx]

**Supplementary Table 2 ⏐ List of siRNAs.**

| **siRNA** | **5' → 3'** | **Company** |
| --- | --- | --- |
| **Gys1** | GGG CGA GGA GCG UAA CUA A | Dharmacon |
|  | CAA CGA CGC UGU CCU CUU U | Dharmacon |
|  | GAA GCU UUA UGA AUC CUU A | Dharmacon |
|  | GAA UCG GCC UCU UCA AUA G | Dharmacon |
| **UGP2** | GGU UCA AGA UUA UCU AAG A | Eurogentec |
|  | GUG GAU CUG UAU AUU CUU A | Eurogentec |
| **SLC35D2** | CCA CAU AAG UGG AUU AUC A | Eurogentec |
|  | GCC ACC AUA AUG AUA CUA U | Eurogentec |
