# Supplementary material for: Sequestration of host metabolism by an intracellular pathogen
Source: eLife. 2016 Mar 16;5:e12552. doi: 10.7554/eLife.12552 (PMC4829429; doi:10.7554/eLife.12552)
Supplement: Supplementary file 3. — DOI: http://dx.doi.org/10.7554/eLife.12552.025 [file elife-12552-supp3.docx]

**Supplementary Table 3 ⏐ List of primers used in qRT-PCR and RT-PCR.**

| **Name** | **Gene** | **Primers** |
| --- | --- | --- |
| **glgA** | CT798**^a^** / CTL0167**^b^** | AATGATTGGAATGCGTTACGG |
|  |  | CGGTAGGTTGTCACTGCTTCC |
| **glgB** | CT866 / CTL0245 | gtgcatcattttggggtagga |
|  |  | ctcgcgatttcaggtgtaagg |
| **glgC** | CT489 / CTL0750 | GCCTTTGCCTCAGAATTTTCC |
|  |  | CCAAACCTGACTTCCATCTCG |
| **glgX** | CT042 / CTL0298 | CTCCCTTTAACCCCCATTTTG |
|  |  | CACGGTAGCATCCATTCCACT |
| **malQ** | CT087 / CTL0342 | cgtcggtctctttcgattttg |
|  |  | gggatcgttcccagatcttct |
| **mrsA** | CT295 / CTL0547 | AGCCCGAGTCTTAAAGCGAAG |
|  |  | CCAAAGGCTCTGGAATCTGAA |
| **uhpC** | CT544 / CTL0806 | cggctttacaggatggttcgc |
|  |  | cggatgcattccatgttggca |
| **omcB** | CT443 / CTL0702 | CTGCAACAGTATGCGCTTGTC |
|  |  | GCTGTTGCTGTTCCTTGGTTC |
| **hctA** | CT743 / CTL0112 | TAAAGCCGCAGCACAAAGAGT |
|  |  | CGAACAGGCTTCTTAGCAGCAG |
| **euo** | CT446 / CTL0706 | TATGCTACACGCATTGGTGCT |
|  |  | GCCTCAAAACCTTCTCTCATGG |
| **slc35d2** | slc35D2 | CTGCCTCTCCTCTACGTTGG |
|  |  | CCCGAGAATAATGGCAAAGA |

**^a^** *Chlamydia trachomatis* strain D/UW-3/CX

**^b^** *Chlamydia trachomatis* strain L2/434/Bu
